# Comparative analysis of gut and skin microbiota from the coastal and non-coastal populations in Tamil Nadu using 16S rRNA gene sequencing: a pilot study

**DOI:** 10.3389/fmicb.2026.1849751

**Published:** 2026-10-26

**Authors:** Rithi Adhilakshmi Thangamani, Jayenth Jayachandran, Ashokkumar Jayavel, Sathish Kumar Malaikani Ponnaiya, Abhijit Mitra, Priyadarshini Shanmugam, Arunkumar Radhakrishnan, Antara Banerjee, Asim K. Duttaroy

**Affiliations:** 1 Department of Pharmacology, Chettinad Hospital and Research Institute, Chettinad Academy of Research and Education, Chennai, India; 2 Ponnaiya’s Code and Genome Pvt Ltd., Madurai, India; 3 Medical Biotechnology Lab, Faculty of Allied and Healthcare Sciences, Chettinad Academy of Research and Education, Chettinad Hospital and Research Institute, Chennai, India; 4 Department of Microbiology, Chettinad Hospital and Research Institute, Chettinad Academy of Research and Education, Chennai, India; 5 Department of Nutrition, Institute of Medical Sciences, Faculty of Medicine, University of Oslo, Norway

**Keywords:** 16S rRNA gene sequencing, coastal habitats, microbiome diversity, relative abundance, taxonomic profiling

## Abstract

**Background:**

The human gut and skin microbiota play a vital role in maintaining physiological balance and immune function. Factors such as age, diet, geography, and genetic factors influence their composition, and disruptions can contribute to metabolic alterations and chronic diseases. Coastal populations are exposed to environmental pollutants and experience disrupted gut microbial balance, impaired epithelial barrier integrity, and possibly dysbiosis-associated inflammatory responses. In this study, we aim to analyze microbiome diversity in coastal and non-coastal populations of Tamil Nadu, India, using 16S rRNA sequencing.

**Methods:**

Fecal and skin swab samples were collected from 75 participants, 57 from coastal regions and 18 from non-coastal regions. DNA was extracted, and using the Illumina MiSeq platform, the 16S rRNA sequencing was performed. The alpha and beta diversity analyses compared the fecal and skin microbiota diversity and composition.

**Results:**

The study revealed that Firmicutes dominated the gut microbiota in both coastal and non-coastal groups, with higher abundances of Bacilli and Clostridia in the coastal group. In skin swabs, Firmicutes and Proteobacteria were predominant, with different clustering patterns observed in coastal samples. Beta diversity analyses revealed overlapping but distinct microbial communities between the two groups.

**Conclusion:**

This pilot study highlights differences in gut and skin microbiota between coastal and non-coastal populations. However, further study with a larger sample size is required to better understand the impact of environmental exposures on human health.

## 1 Introduction

The gut microbiome significantly impacts the gastrointestinal tract by influencing digestion, nutrient metabolism, maintenance of intestinal barrier function, and host immune regulation (Valdes et al., 2018). Recent studies have provided evidence that an interaction between the intestinal microbiome and immune responses, neurological conditions, and drug compounds is involved in maintaining general health (Sasso et al., 2023). Various factors, including age, dietary patterns, geographic location, and an individual’s genetic composition, impact gut microbiome composition (Hou et al., 2022). Alterations in the gut microbiome have been associated with changes in human health and metabolism, highlighting that the relationships between bacterial species and human metabolism remain to be determined (Wu et al., 2020). However, recent techniques examining microbiome diversity have established links between particular intestinal bacteria and human health (Wensel et al., 2022). Environmental exposure and geographical variation may also contribute to differences in gut microbial composition, particularly among individuals exposed to different environmental factors and distinct lifestyles (Yuan et al., 2026). However, limited research has compared alterations in the gut microbiome between coastal and non-coastal populations.

The composition of the healthy skin microbiota plays an important role in maintaining overall skin health and influences the development, epidemiology, and severity of skin diseases (Skowron et al., 2021). Populations in contact with polluted water can be affected by a wide range of pathogens, including viruses, pathogenic bacteria, and intestinal parasites, that inhabit sewage and stormwater (Nielsen and Jiang, 2019). The skin microbiome is a diverse collection of microorganisms, including bacteria, fungi, and viruses, that colonize the surface of the human skin (Schommer and Gallo, 2013) and also plays an important role in the regulation of skin homeostasis and the function of the human immune system (Li et al., 2025). Numerous factors, including age, personal hygiene, environmental factors, geographic location, and occupational exposure, affect the overall composition of skin microbial diversity (Mennitti et al., 2025). A recent study by Potbhare et al. (2022) highlights that the composition of the skin microbiota may differ significantly due to host-associated traits, dietary patterns, environmental exposures, and geographic variations. Firmicutes, Proteobacteria, and Actinobacteria were significantly more prevalent in skin samples from Indian individuals (Potbhare et al., 2022). However, geographic-based differences in skin microbial communities among Indian families were further reported by Potbhare et al. (2025). These findings indicate that the composition of the skin microbiota across different human populations may be significantly altered by environmental factors (Potbhare et al., 2025). Skin dysbiosis has been implicated in several dermatological conditions, such as acne, atopic dermatitis, psoriasis, and wound infections (Borrego-Ruiz and Borrego, 2024). Although there is an increasing interest in how environmental factors may affect the human microbiome, few studies have compared the alterations in the skin microbiota (Chaudhari et al., 2020; Potbhare et al., 2025).

Pharmaceutical chemicals and other inorganic and organic pollutants that may impact the microbial population are frequently found in coastal sediments near wastewater treatment plants and drains (Habibi et al., 2023; Masoner et al., 2019). Nielsen and Jiang's (2019) highlight that swimming in polluted ocean water disrupts the skin’s microbiome, which may permit opportunistic pathogens to colonize the host transiently, although further research is needed to evaluate the risk of infection (Nielsen and Jiang, 2019). Gut microbial stability is also affected by environmental exposures in coastal regions (See et al., 2025). Environmental contaminants, including heavy metals, organic pollutants, and contaminated water sources, may contribute to gut dysbiosis through mechanisms such as oxidative stress, modulation of cytokine responses, reduced microbial diversity, and impairment of epithelial barrier integrity (Chiu et al., 2020). Although the 16S rRNA gene is highly conserved across bacterial species, it also contains variable regions that aid in recognizing various bacterial types (Durazzi et al., 2021). Additionally, there is increasing evidence associating alterations in the diversity and activity of intestinal microbes with the emergence of diseases like neurological disease (Kumar et al., 2023), type 2 diabetes (Zhou et al., 2019), cancer (Adekunle et al., 2024), and inflammatory bowel disease (Lloyd-Price et al., 2019). Few previous studies on the human gut microbiome have linked gut bacterial composition to several conditions, such as type 2 diabetes and inflammatory bowel disease (De Vos et al., 2022).

Indian population comprises genetically heterogeneous groups with diverse dietary habits and geographical distributions (Samir et al., 2018). So, human microbiome composition varies considerably among individuals and is influenced by environmental and lifestyle-related factors. We hypothesize that chronic exposure to contaminants in marine environments may alter the composition and diversity of the gut and skin microbiomes. In this preliminary study, we conducted an initial analysis of the gut and skin microbiomes in 75 participants, comprising 57 participants from coastal and 18 from non-coastal regions. Using 16S rRNA gene sequencing, we analyzed the microbial composition, taxonomic distribution, and relative abundance in fecal and skin swab samples collected from coastal and non-coastal populations in Tamil Nadu, India.

## 2 Materials and methods

### 2.1 Study design and population

This study involves 75 participants from Tamil Nadu, India, including 57 participants from the eastern coastal region of Tamil Nadu, from areas including Sadras, Kalpakkam, Pudupattinam, Pattinampakkam, Kasimedu, Venpurusham, and Ennore, and 18 participants from the non-coastal region, Ariyalur. All the participants belong to the South Indian population of Asian Ethnicity. Fecal and skin swab samples were collected from the health camp conducted by Chettinad Hospital and Research Institute, Kelambakkam, Chennai, in both coastal and non-coastal regions of Tamil Nadu. Participants underwent a thorough physical examination and a questionnaire-based interview to collect demographic data, medical history, and diagnostic details. The coastal population included individuals both with and without skin diseases to evaluate the possible influence of coastal environmental exposure on gut and skin microbiota composition. Differences in microbiota composition between healthy and diseased skin may arise from variations in skin barrier integrity, inflammation, immune responses, and local environmental conditions. Accordingly, coastal participants were sub-grouped by the presence or absence of skin disease, whereas non-coastal participants were used for comparison, as they had no visible clinical skin manifestations.

#### 2.1.1 Inclusion and exclusion criteria

The study was conducted among non-coastal and coastal populations inhabiting the East Coast of Tamil Nadu, India. Participants aged 18 years and above were included in the study. Individuals from the coastal population with and without skin diseases were included to evaluate variations in skin and gut microbiota composition. Individuals who had resided in coastal areas for less than 5 years, had recent antibiotic, antifungal, or topical medication usage, chronic gastrointestinal disorders, severe systemic illness, and recent hospitalization were excluded from the study. The coastal population was further categorized based on the presence or absence of skin disease. The non-coastal population was also examined for skin disease. However, none of the non-coastal participants had skin disease; therefore, they were included in the baseline comparison. A total of 78 samples (feces and skin swabs) were collected from the study participants. In the coastal group, 30 fecal samples and 30 skin swab samples were initially collected. Following the sequencing quality control check, two coastal skin swab samples were excluded, resulting in 30 fecal and 28 skin swab samples. In the non-coastal group, 10 fecal samples and 10 skin swab samples were collected, and all passed quality control. Among these, three participants in the coastal group and two participants in the non-coastal group provided both fecal and skin swab samples. The remaining fecal and skin swab samples were collected from different individuals. Demographic and lifestyle-related information, including age, sex, occupation, dietary habits, smoking habits, and alcohol consumption, were collected during participant enrolment. Most participants were in the 19–59 age group and predominantly followed a mixed South Indian rice-based dietary pattern.

#### 2.1.2 Ethical approval

This study received approval from the Institute Human Ethics Committee (Ref No: IHEC-II/0098/21) at Chettinad Hospital and Research Institute, Chettinad Academy of Research and Education, India. All participants provided written informed consent before enrollment in this study.

### 2.2 Methodology

Out of 75 participants, 57 were from the east coast of Tamil Nadu and 18 were from non-coastal areas of Tamil Nadu. From the study participants, fecal samples were collected using sterile culture containers, and skin swab samples were obtained using sterile culture swab tubes. Totally, 30 fecal and 28 skin swab samples from the coastal population and 10 fecal and 10 skin swab samples from the non-coastal population were analyzed for 16S rRNA sequencing. Skin swab samples were collected from the forearm or from affected/wounded areas using sterile swabs moistened with 0.9% Sodium Chloride (NaCl). Among the coastal participants, skin swabs were collected from three main groups: (1) fishermen and fisherwomen with skin diseases such as eczema, tinea, and fissures; (2) fishermen and fisherwomen without any skin diseases; and (3) coastal residents (not actively going fishing) who had skin diseases. All collected samples were transported to the laboratory using an ice pack and brought to the Chettinad Academy of Research and Education within 4–5 h. Samples were immediately stored at −80 °C until Deoxyribonucleic acid (DNA) extraction was performed.

#### 2.2.1 DNA extraction

A small amount (approx. 200 mg) of fecal samples was aliquoted into two 2 mL microcentrifuge tubes, homogenized in 1 mL of phosphate-buffered saline (PBS), and centrifuged at 5,000 rpm for 5 min at room temperature. Genomic DNA (gDNA) was isolated from stool samples using the phenol-chloroform-isoamyl alcohol (PCI) method (Srirungruang et al., 2022). After extraction, the gDNA was quantified using NanoDrop Lite (ThermoFisher Scientific). In addition, gDNA was isolated from skin swab samples using the QIAamp DNA Microbiome Kit (QIAGEN, USA), according to the manufacturer’s instructions. All extracted DNA samples were quantified using the NanoDrop Lite spectrophotometer at 260/280 nm (A260/280) to determine DNA concentration and estimate purity. The NanoDrop is calibrated with the blank solution (Nuclease-free water). After assessing DNA quality and quantity, the samples were stored at −80 °C until 16S rRNA sequencing was performed on the Illumina MiSeq platform. No positive controls, such as mock communities, or negative controls were included during the DNA extraction, library preparation, or sequencing workflow.

#### 2.2.2 16S rRNA gene amplicon processing and analysis

High-quality gDNA samples were subjected to a quality check using the Implen NP80 spectrophotometer (Implen, Germany), and the A260/280 ratio was recorded, followed by a general 16S rRNA gene PCR for amplification of the V3-V4 region. Samples that failed in the PCR were excluded, irrespective of whether they qualified the spectrophotometric QC. This was done to ensure the success of library preparation. Samples were sequenced using the Illumina MiSeq instrument. The Illumina MiSeq sequencer was used to determine the order of nucleotides in the V3-V4 region (341F/785R) of DNA or RNA. The Qubit fluorometer was used to quantify the polymerase chain reaction (PCR)-positive samples by using the double-stranded deoxyribonucleic acid (dsDNA) kit from ThermoFisher Scientific, Inc. Samples were diluted to a 1 ng/μL concentration in Low TE buffer and used to prepare a library of the 16S rRNA gene target for sequencing of the V3-V4 region. Library preparation was performed according to the Illumina protocols, as described in Fadrosh et al. (2014), for 16S metagenomics using a phased primer strategy. Indexed libraries were pooled and purified to create single libraries that were purified and confirmed for their equimolarity to ensure they were prepared to a concentration of 10 nM before being analyzed using an Agilent TapeStation 4,200, with the high-sensitivity D1000 screentape (Agilent Technologies, USA) and a Sensitive Fluorescence Assay (ThermoFisher Scientific) for measuring the concentration of dsDNA on a Qubit fluorometer. The pooled library was then analyzed on an Illumina MiSeq platform using a v2 reagent kit and 2 × 250-base chemistry, as per the manufacturer’s protocol. Results from each of the three runs produced demultiplexed reads, which were directly sequenced to produce FastQ files. Finally, a comparison of the coastal and non-coastal 16S bacterial sequences was performed based on this analysis.

### 2.3 Data analysis

FastQC (version 0.11.3) was used to evaluate the integrity of the data from Illumina MiSeq sequencing (Andrews, 2010), and the DADA2 software program (version 1.3.1) (Callahan et al., 2017). The DADA2 pipeline encompasses read filtering and trimming, dereplication, error profiling, sample inference, merging of paired-end reads, construction of the sequence table, removal of chimeras, and taxonomy assignment. Diversity analysis was performed using the core diversity analyses script from QIIME (version 1.9.0) (Caporaso et al., 2010). Core diversity analyses calculated four alpha diversity measures (observed species, Chao, Shannon Index, Simpson Index) and two beta diversity measures (Bray-Curtis and Unweighted UniFrac Principal Coordinate Analysis (PCoA)). Comparisons were made at the taxonomic levels of phylum, class, and order between the different sample groups tested. Figures were made using ggplot2 in RStudio (Mukhopadhya et al., 2022). PCoA plots were visualized using EMPeror. Relative abundance analysis was performed in R (v4.5.1) using the DADA2 and phyloseq pipelines for amplicon sequence data processing and visualization. Taxonomic assignment was performed against the SILVA database, and relative abundance plots were generated using phyloseq visualization functions in RStudio. The plotted data represent normalized abundance values (as a percentage of total reads per sample) at the phylum level. Taxa without phylum-level annotations were excluded before phylum-level grouping, and no prevalence or minimum abundance cutoff was applied. Functional profiling of metabolic pathways was conducted using PICRUSt2 (Phylogenetic Investigation of Communities by Reconstruction of Unobserved States 2) as described by Beghini et al. (2021).

### 2.4 Statistical analysis

Alpha-diversity metrics, representing within-sample diversity, were calculated and analyzed using GraphPad Prism version 8.0, and differences between the coastal and non-coastal groups were assessed using the Mann–Whitney non-parametric test, with *p*-values adjusted for multiple comparisons across diversity metrics using the Benjamini–Hochberg (BH) correction; q-values ≤ 0.05 were considered statistically significant. For beta diversity, differences in microbial community composition between the coastal and non-coastal groups were evaluated using Permutational Multivariate Analysis of Variance (PERMANOVA) with 999 permutations.

## 3 Result

### 3.1 Characteristics of the study participants

Overall, 78 samples were collected from the 75 study participants: 30 fecal and 28 skin samples from coastal regions, and 10 fecal and 10 skin samples from non-coastal regions. The socio-demographic details of study participants, including age, sex, occupation, dietary habits, smoking habits, and alcohol consumption, are summarized in Supplementary Table 1. Gender distribution varied slightly between the two populations, with a higher proportion of females in the coastal population than in the non-coastal population. Among the fecal samples analyzed, 100% of the non-coastal participants and 80% of the coastal participants were within the 19–59 years’ age group. Similarly, among the skin swab samples analyzed, 100% of non-coastal participants and 71.4% of coastal participants were in the 19–59 age group. Participants from both populations predominantly followed a mixed South Indian rice-based dietary pattern. Regular seafood consumption was more common among coastal participants, whereas non-coastal participants showed more occasional seafood intake. Occupational differences were also observed, with fishing-related occupations predominantly identified among coastal participants. Additionally, alcohol consumption was more prevalent among coastal participants than non-coastal participants.

### 3.2 16S rRNA gene sequencing-based microbial profiling

DNA extracted from fecal and skin swab samples from both coastal and non-coastal groups was used for 16S rRNA gene sequence analyses. A total of 528,957 reads in skin swabs and 1,577,705 reads in feces have high-quality 16S rRNA gene sequence read pairs that were obtained from different sample types sequenced (feces and skin swabs) following quality filtering. A total of 34 taxa were identified in fecal samples from the non-coastal group, and 43 taxa in the coastal group. In skin swab samples, 41 taxa were identified in the non-coastal group and 137 taxa in the coastal group. The relative abundance of these taxa in each sample was determined using DADA2 analysis (Table 1).

**Table 1:** Overview of sequencing data of the fecal and skin swab samples from the coastal and non-coastal population.

Sequencing metrics	Fecal samples		Skin swab samples	
	Non-coastal	Coastal	Non-coastal	Coastal
Total reads	310,200	1,267,505	34,340	494,617
Total taxa identified	34	43	41	137
Total phyla identified	10	10	10	23

### 3.3 Microbiome diversity of the samples from coastal and non-coastal regions

#### 3.3.1 Microbiome diversity of the fecal samples

After quality control, operational taxonomic unit (OTU)-level diversity was quantified through standard α- and β-diversity metrics to compare gut microbiota between the two populations. Rarefaction curves for every library reached clear plateaus (Figure 1a), confirming that read depth was sufficient to capture the inhabiting genera. α-Diversity was assessed with the Observed-species, Chao1, Shannon, and Simpson indices. Species counts ranged from 7 OTUs in one of the non-coastal samples to 91 OTUs in the sample with the highest species richness among the coastal samples (Figure 1b). Outliers were detected for Observed species (one coastal, one non-coastal), Chao1 (one coastal), Shannon (two coastal, one non-coastal), and Simpson (four coastal, one non-coastal) indices. After exclusion of the identified outliers from the coastal and non-coastal groups, differences in alpha diversity were assessed using the Mann–Whitney U test; *p*-values were adjusted for multiple comparisons using the Benjamini–Hochberg (BH) correction. No statistically significant differences were observed (Supplementary Table 2).

**Figure 1:**
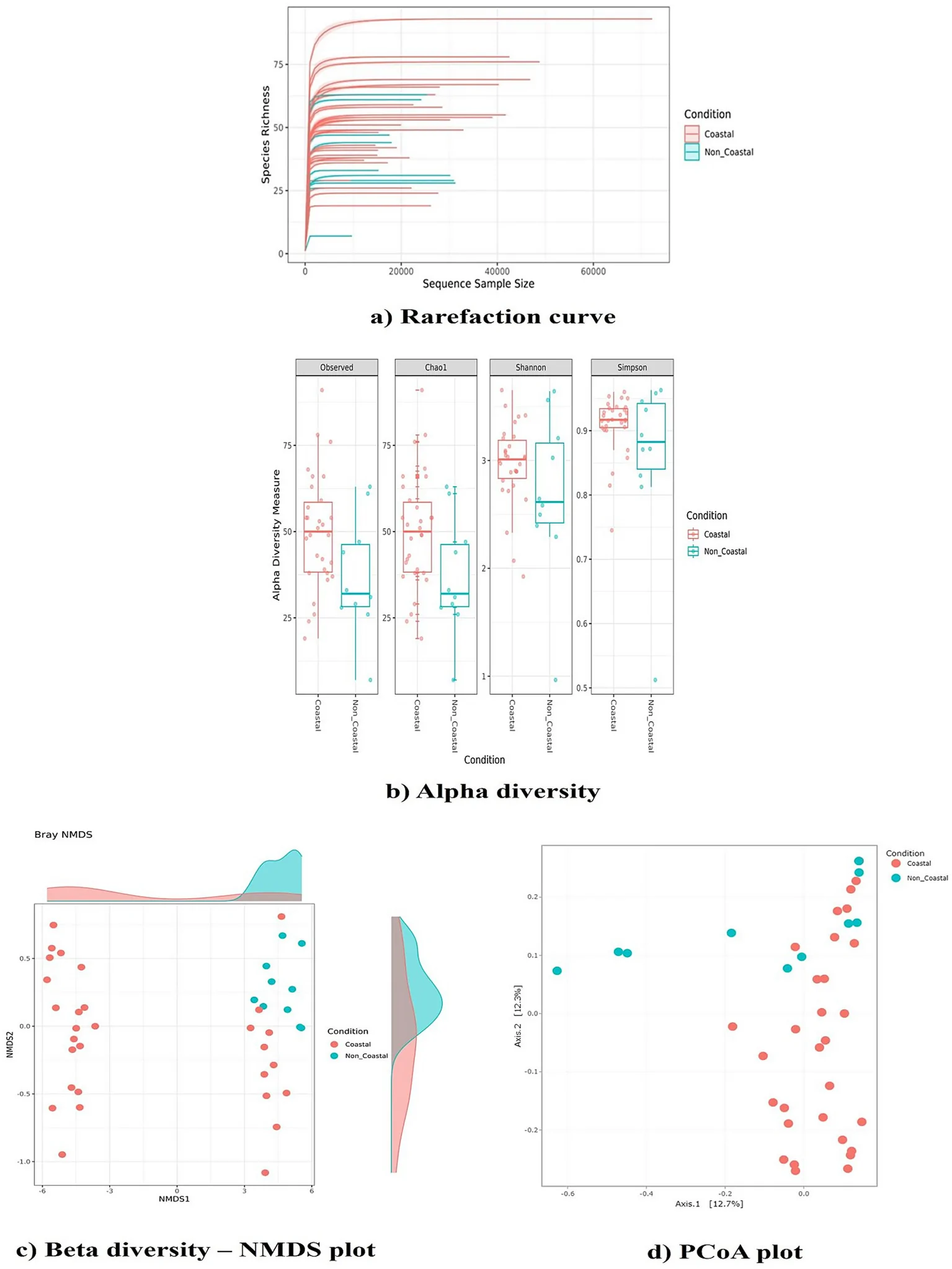
Analysis of microbial diversity on fecal samples with coastal and non-coastal control groups. **(a)** rarefaction curves, **(b)** alpha diversity of the fecal samples (Observed species, Chao1 richness index, Shannon index, and Simpson index), **(c)** analysis of beta diversity in the coastal and non-coastal control groups – Bray-Curtis NMDS plots, **(d)** PCoA analysis based on Unifrac distance matrices (blue color: non-coastal group and red color: coastal group).

Bray-Curtis Non-metric Multidimensional Scaling (NMDS) revealed that some coastal and non-coastal samples formed discrete clusters, while a subset of coastal and non-coastal samples showed partial overlap (Figure 1c). Similarly, unweighted UniFrac PCoA likewise placed most coastal and non-coastal samples near the AXIS1/AXIS2 origin, with partial overlap, although a few non-coastal samples occupied distinct positions and did not form clusters (Figure 1d). Together, these results indicate substantial differences in community composition between coastal and non-coastal gut microbiomes. To statistically evaluate these patterns, PERMANOVA based on Bray–Curtis dissimilarity with 999 permutations was performed. The analysis revealed a significant difference in microbial community composition between the coastal and non-coastal groups (*F* = 2.24, R^2^ = 0.057, *p* = 0.001). The homogeneity of the multivariate dispersion test was significant (*F* = 18.55, *p* < 0.001), indicating differences in within-group dispersion between the study groups. Therefore, the PERMANOVA result for fecal samples may be influenced by differences in group dispersion and community composition.

#### 3.3.2 Microbiome diversity of the skin swab samples

Among the skin swab samples collected from both populations, both diseased and healthy skin samples were included in the coastal population. 15 (53.6%) of participants had normal skin, 7 (25%) had eczema, 5 (17.9%) had tinea, and 1 (3.5%) had dry skin, which were observed in the coastal population. However, among non-coastal participants, 10 (100%) exhibited normal skin, and no cases of eczema, tinea, or dry skin were observed. The most commonly observed skin conditions among coastal participants were eczema and tinea (Supplementary Table 3). OTU-based α- and β-diversity metrics were then applied to compare skin microbiome composition. Rarefaction curves plateaued for every sample (Figure 2a), indicating that read depth was sufficient to capture genus-level diversity in both populations. Richness and evenness were quantified with Observed OTUs, Chao1, Shannon, and Simpson indices (Figure 2b). Alpha diversity was initially compared between all skin swab samples from coastal and non-coastal groups using the Mann–Whitney U test. In this analysis, outliers were detected for Observed species and Chao1 (two coastal), Shannon (one non-coastal), and Simpson (four coastal, one non-coastal) indices. After exclusion of the identified outliers, statistically significant differences were observed for the Observed OTUs, Chao1, Shannon, and Simpson indices after adjustment for multiple comparisons using the Benjamini–Hochberg (BH) correction (Supplementary Table 4a). In the coastal group, participants with normal skin and diseased skin were observed. Furthermore, to minimize the influence of skin disease, alpha diversity was also assessed using only clinically normal skin samples collected from participants in both the coastal and non-coastal groups. In this analysis, one non-coastal sample was flagged as an outlier in Shannon, while one non-coastal sample and two coastal samples were identified as outliers for Simpson and excluded before performing the Mann–Whitney U test. Significant differences between the healthy coastal and healthy non-coastal groups were observed after BH correction (Supplementary Table 4b). Bray-Curtis NMDS (Figure 2c) revealed partial separation between coastal and non-coastal samples, with greater dispersion among coastal samples. Unweighted UniFrac PCoA (Figure 2d) formed distinct clusters of coastal and non-coastal samples; also, some coastal and a few non-coastal samples occupied borderline positions, away from the main cluster centered near AXIS1/AXIS2 ~ 0.2. These patterns highlight variability in the skin microbiota among coastal and non-coastal populations. To statistically evaluate these patterns, PERMANOVA based on Bray–Curtis dissimilarity with 999 permutations was performed using the vegan package in R. The analysis revealed a significant difference in microbial community composition between the coastal and non-coastal groups (*F* = 1.29, R^2^ = 0.035, *p* = 0.041). The skin samples showed no significant difference in multivariate dispersion between the coastal and non-coastal groups (*F* = 3.72, *p* = 0.062), indicating homogeneity within-group dispersion.

**Figure 2:**
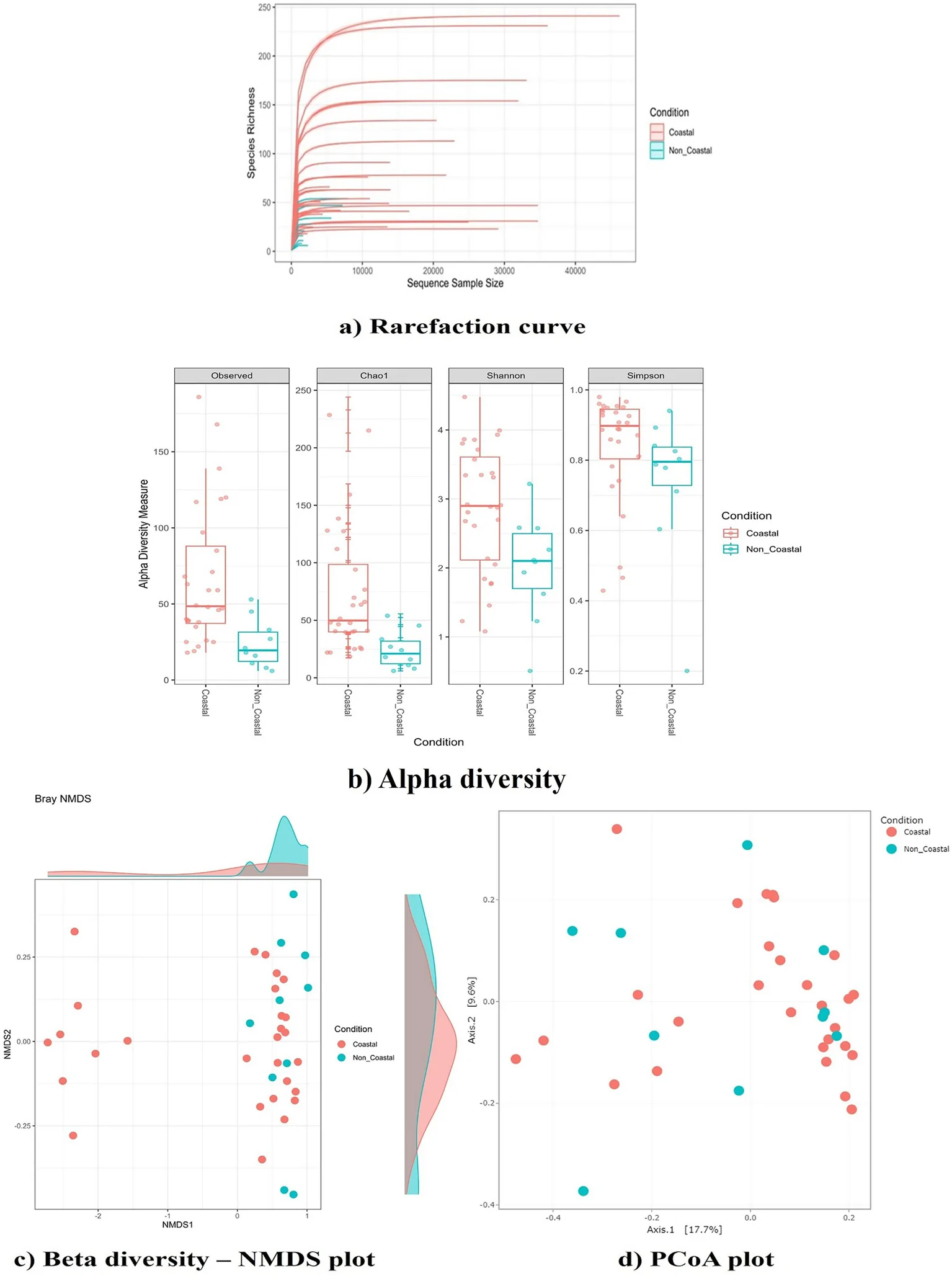
Analysis of microbial diversity on skin swab samples with coastal and non-coastal control groups. **(a)** rarefaction curves, **(b)** alpha diversity of the skin swab samples (Observed species, Chao1 richness index, Shannon index, and Simpson index), **(c)** analysis of beta diversity in the coastal and non-coastal control groups – Bray-Curtis NMDS plots, **(d)** PCoA analysis based on Unifrac distance matrices (blue color: non-coastal group and red color: coastal group).

### 3.4 Comparison of microbial profiles between non-coastal and coastal regions

#### 3.4.1 Microbiome composition in fecal samples

Figure 3 illustrates the average diversity of the gut microbiome at the phylum-to-order taxonomic levels for both coastal and non-coastal groups. At the phylum level, fecal samples from the coastal group showed a relatively higher abundance of Firmicutes, whereas the non-coastal group was predominantly enriched with Bacteroidota. Regional and geographical influences appear to play a crucial role in affecting the taxonomic composition of the microbiota. Among Firmicutes, Clostridia and Bacilli showed greater prevalence in the coastal group, followed by Negativicutes, compared to the non-coastal group. This finding indicates a unique microbiota composition associated with coastal regions. A higher abundance of Lachnospirales, Oscillospirales, and Clostridia UCG-014 was noted within the Clostridia class among coastal individuals. Additionally, the Bacilli class, Erysipelotrichales, and Lactobacillales were more prevalent in the coastal group. Furthermore, the coastal group exhibited a higher abundance of Selenomonadales in the class Negativicutes than the non-coastal group. Overall, the findings show that the gut microbiota composition of coastal and non-coastal populations differs with regional environmental, dietary, and lifestyle variables that may influence the diversity of particular taxa.

**Figure 3:**
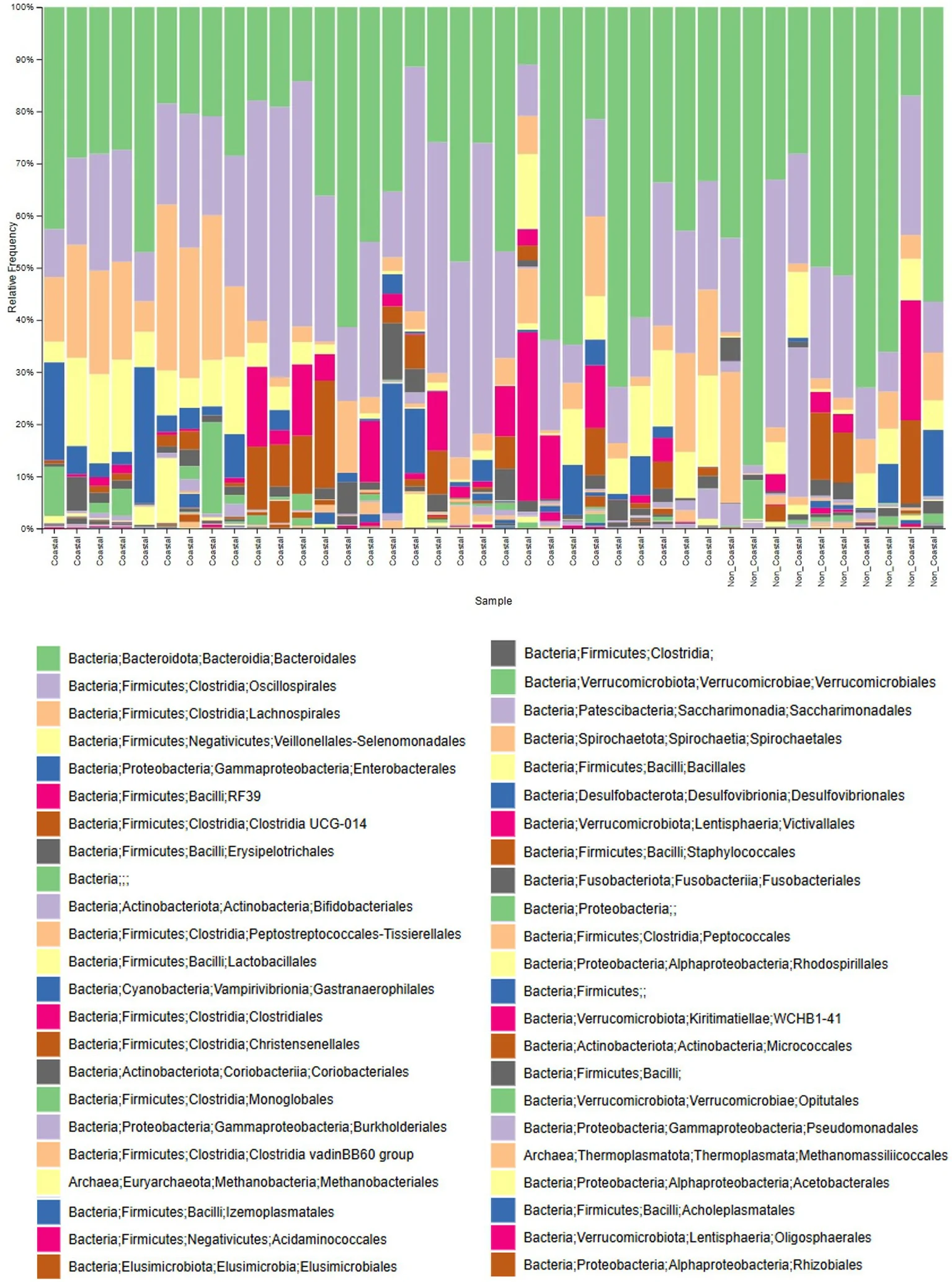
Distribution of the differential microbiota at the phylum-to-order levels between the coastal and non-coastal groups of fecal samples.

#### 3.4.2 Microbiome composition in skin swab samples

Figure 4 illustrates the mean relative abundance of the microbiome in the skin swab samples in coastal and non-coastal groups. In skin swab samples from both groups, Firmicutes was the most common phylum at the phylum level, followed by Proteobacteria, highlighting the primary microbial phyla inhabiting the skin. The coastal group had a greater abundance of Bacilli and Clostridia than the non-coastal group, based on the class level. Within the Proteobacteria phylum, the classes Gammaproteobacteria and Alphaproteobacteria were more abundant in the coastal group compared to the non-coastal group, suggesting an alteration in microbial community composition associated with coastal living.

**Figure 4:**
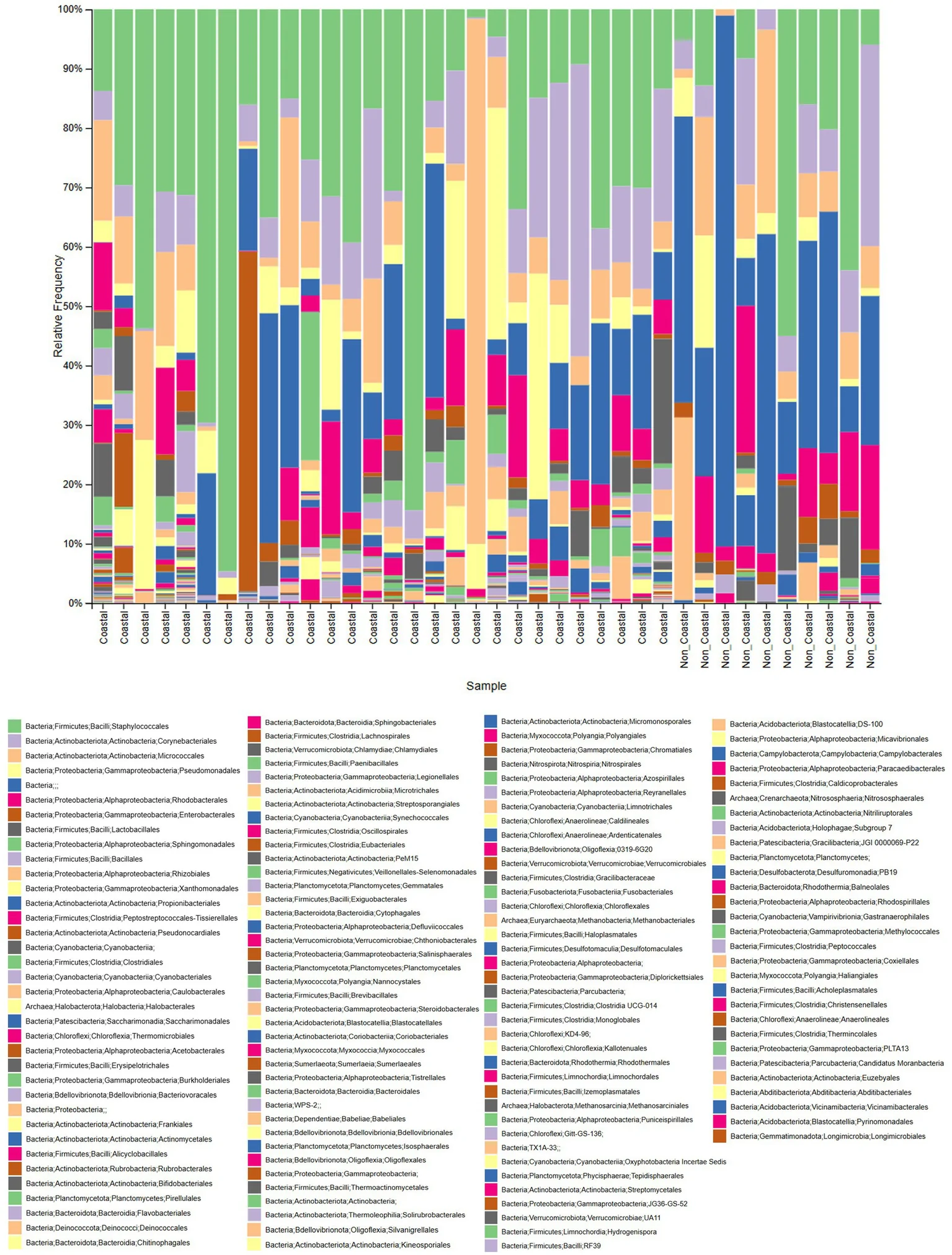
Distribution of the differential microbiota at the phylum-to-order levels between the coastal and non-coastal groups of skin swab samples.

Further investigation at the order level revealed some differences between the two groups. In the Clostridia order of the Clostridia class, the Peptostreptococcales-Tissierellales and Clostridiales had higher abundance in coastal individuals. In contrast, within the Bacilli order for the Bacilli class, abundance was high in Staphylococcales, Bacillales, and Lactobacillales for coastal individuals. In addition, within the Gammaproteobacteria group of bacteria, the Pseudomonadales and Enterobacterales orders were highly present in coastal individuals compared to non-coastal individuals. Furthermore, within the Proteobacteria phylum, the Alphaproteobacteria class, the Rhodobacterales and Sphingomonadales orders showed higher abundance in coastal individuals than in non-coastal individuals.

Figure 5 illustrates the top five abundant bacterial phyla present in both fecal and skin swabs obtained from both coastal and non-coastal populations. The most abundant bacteria have been selected based on their abundance across all samples from coastal and non-coastal populations. The most abundant bacterial phyla in both fecal and skin swabs are Firmicutes, Actinobacteriota, and Proteobacteria.

**Figure 5:**
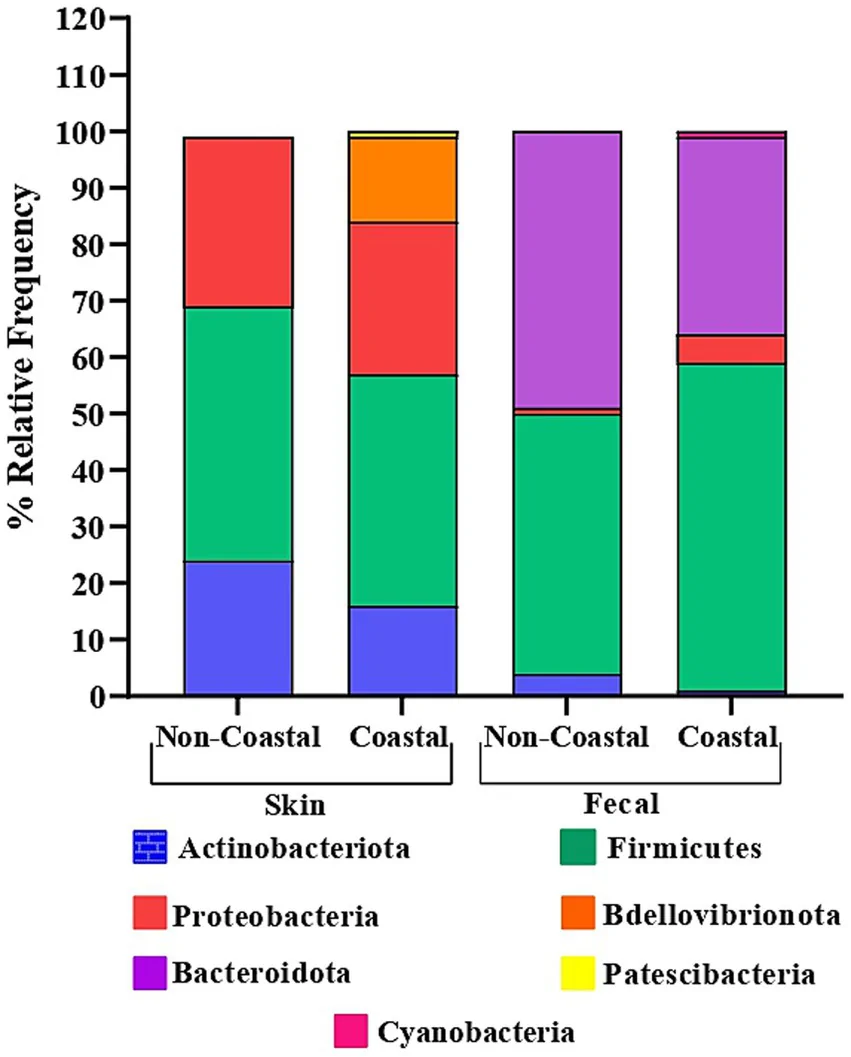
Comparison of Top 5 Phyla in Gut and Skin Microbiota between coastal and non-coastal groups.

### 3.5 Relative abundance of bacterial taxa at the genus level

The relative abundance of the dominant bacterial taxa identified in fecal and skin swab samples from coastal and non-coastal individuals is presented in Figure 6. In fecal samples, Bacteroidales was the predominant bacterial taxon in most individuals from both coastal and non-coastal groups, followed by Oscillospirales, Veillonellales-Selenomonadales, Lachnospirales, and Clostridia UCG-014 in non-coastal groups, whereas Oscillospirales, Lachnospirales, Veillonellales-Selenomonadales, and Enterobacterales were predominant in coastal groups. However, the relative abundance of these bacterial taxa varied among individual samples. In skin swab samples, Staphylococcales was the predominant in most of the individuals from both coastal and non-coastal groups, followed by Micrococcales, Oligoflexales, Pseudomonadales, and Rhizobiales in the non-coastal groups and Corynebacteriales, Micrococcales, Pseudomonadales, and Rhodobacterales in the coastal groups. Overall, the observed differences in the abundance of dominant bacterial taxa across fecal and skin microbiota indicate substantial variation in microbial community composition among individuals, which may reflect the influence of environmental conditions and lifestyle factors on microbiota composition.

**Figure 6:**
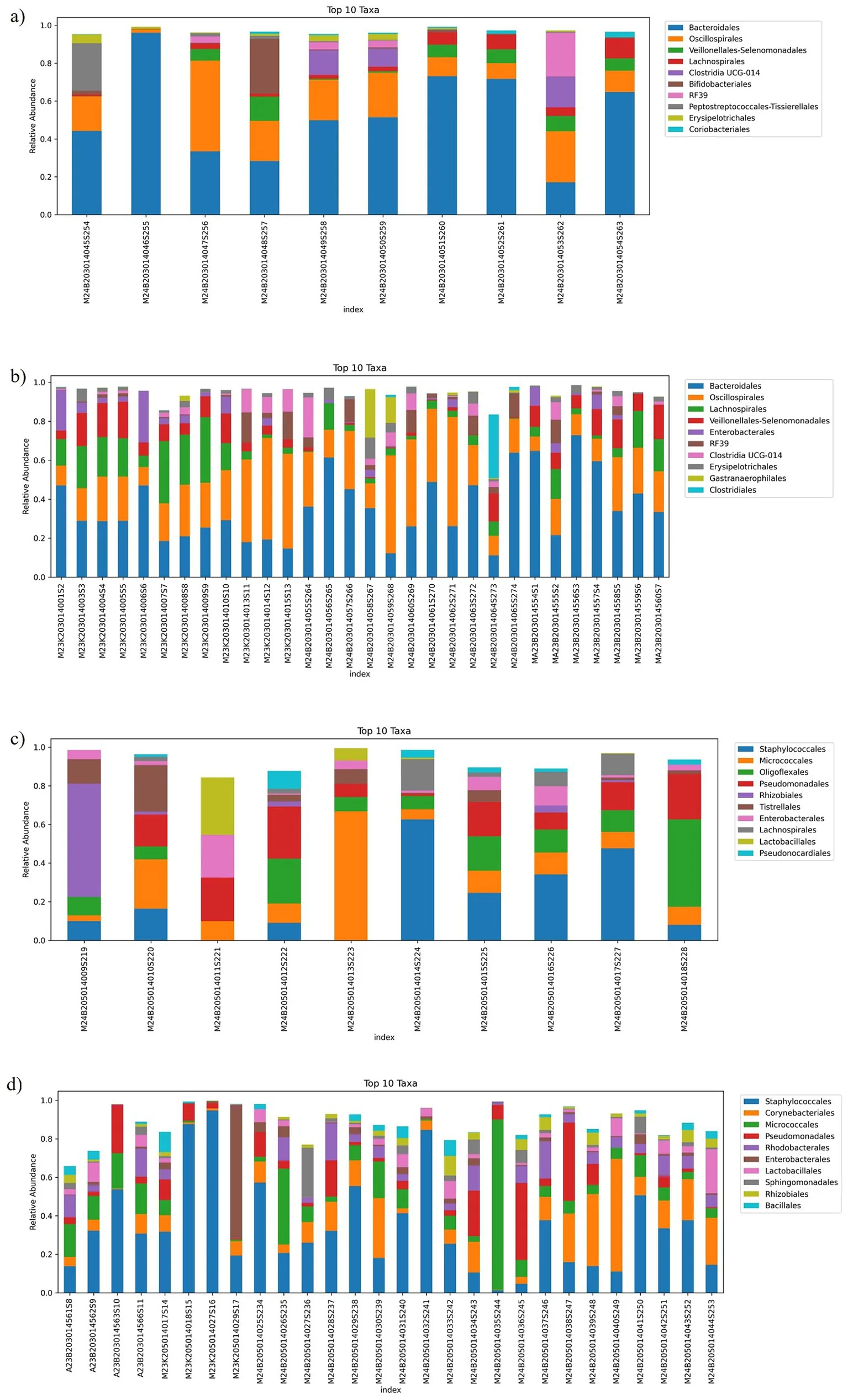
Relative abundance of dominant bacterial taxa in fecal and skin swab samples from coastal and non-coastal populations. **(a)** Non-coastal fecal samples, **(b)** Coastal fecal samples, **(c)** Non-coastal skin swab samples, and **(d)** Coastal skin swab samples.

### 3.6 Comparative analysis of phylum*-*level relative abundance between coastal and non-coastal groups by fecal and skin samples

In the fecal samples, the dominant phyla in the coastal and non-coastal samples were Firmicutes and Bacteroidota, but their relative proportions differed significantly. In the coastal fecal samples, the dominant phylum was Firmicutes, followed by Bacteroidota, whereas the non-coastal fecal samples showed a predominance of Bacteroidota followed by Firmicutes. Low relative abundances of Proteobacteria and Cyanobacteria were observed in the coastal group, indicating lower compositional diversity, which may be due to the effects of the coastal environment and diet. Similarly, the skin samples were dominated by Proteobacteria, with less abundance of Actinobacteriota and Firmicutes. Hence, the comparative analysis reveals differences in microbiome composition between the coastal and non-coastal groups, as shown in Figure 7. In fecal samples, diversity was between Firmicutes and Bacteroidota, and in skin samples, dominance was in Proteobacteria, especially in non-coastal samples. Thus, these results imply that environmental factors may affect the microbiome composition of both gut and skin.

**Figure 7:**
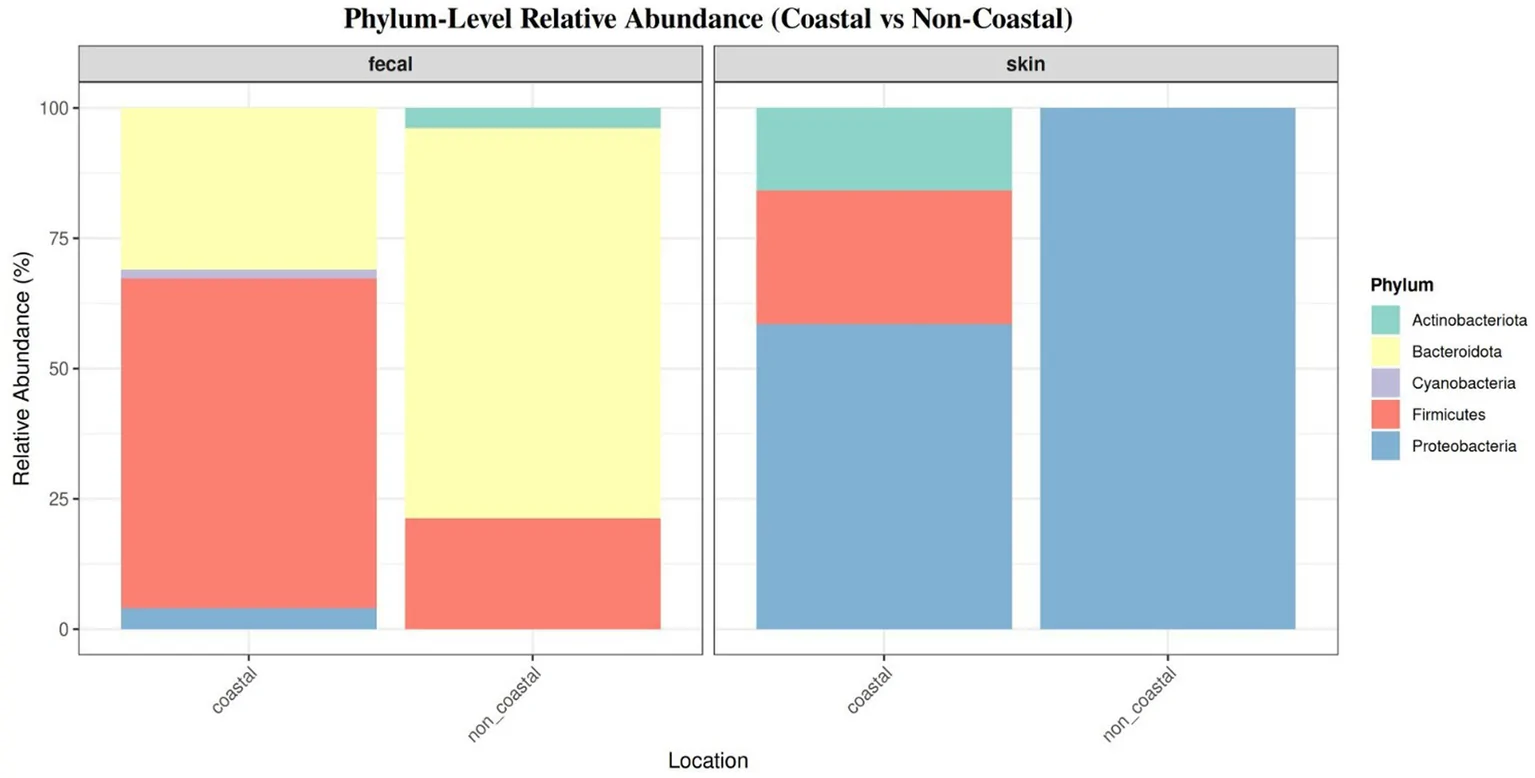
Comparison of phylum-level relative abundance between coastal and non-coastal groups of fecal and skin swab samples.

### 3.7 Differential abundance analysis of gut microbiome of coastal and non-coastal participants

To identify abundant bacterial taxa in fecal samples from coastal and non-coastal groups, we performed linear discriminant analysis effect size (LEfSe) (Wang et al., 2019), and the results were visualized as a cladogram in Figure 8. This analysis was based on 47 Amplicon sequence variants and used linear discriminant analysis scores to highlight taxonomic hierarchies from the phylum to the species level. Nodes and branches are color-coded to represent the group in which specific taxa were significantly enriched, red for coastal and purple for non-coastal populations. The coastal group exhibited a greater enrichment of taxa within the phyla Firmicutes and Proteobacteria. In the Firmicutes phylum, the Clostridia class, the Clostridiales order, especially the Lachnospiraceae family, and genera such as *Coprococcus* and *Lachnospira* are predominant, and in the Proteobacteria phylum, including the Gammaproteobacteria class, the Succinivibrionaceae family, and the *Succinivibrio* genus are predominant. The non-coastal population showed significant enrichment of taxa from the phylum Bacteroidota, including the class Bacteroidia, and the genus *Bacteroides*. The observed differential taxonomic distribution may reflect geographical and environmental variation between study populations. However, lifestyle-associated factors and correlation with environmental exposure to heavy metals or other pollutants in the selected populations were not comprehensively evaluated in the present study. Therefore, the identified microbial differences should be interpreted as preliminary associations rather than definitive coastal-specific effects. For skin swab samples, no significant markers were found, as no microbial taxa or features in the dataset were statistically significantly different between coastal and non-coastal groups.

**Figure 8:**
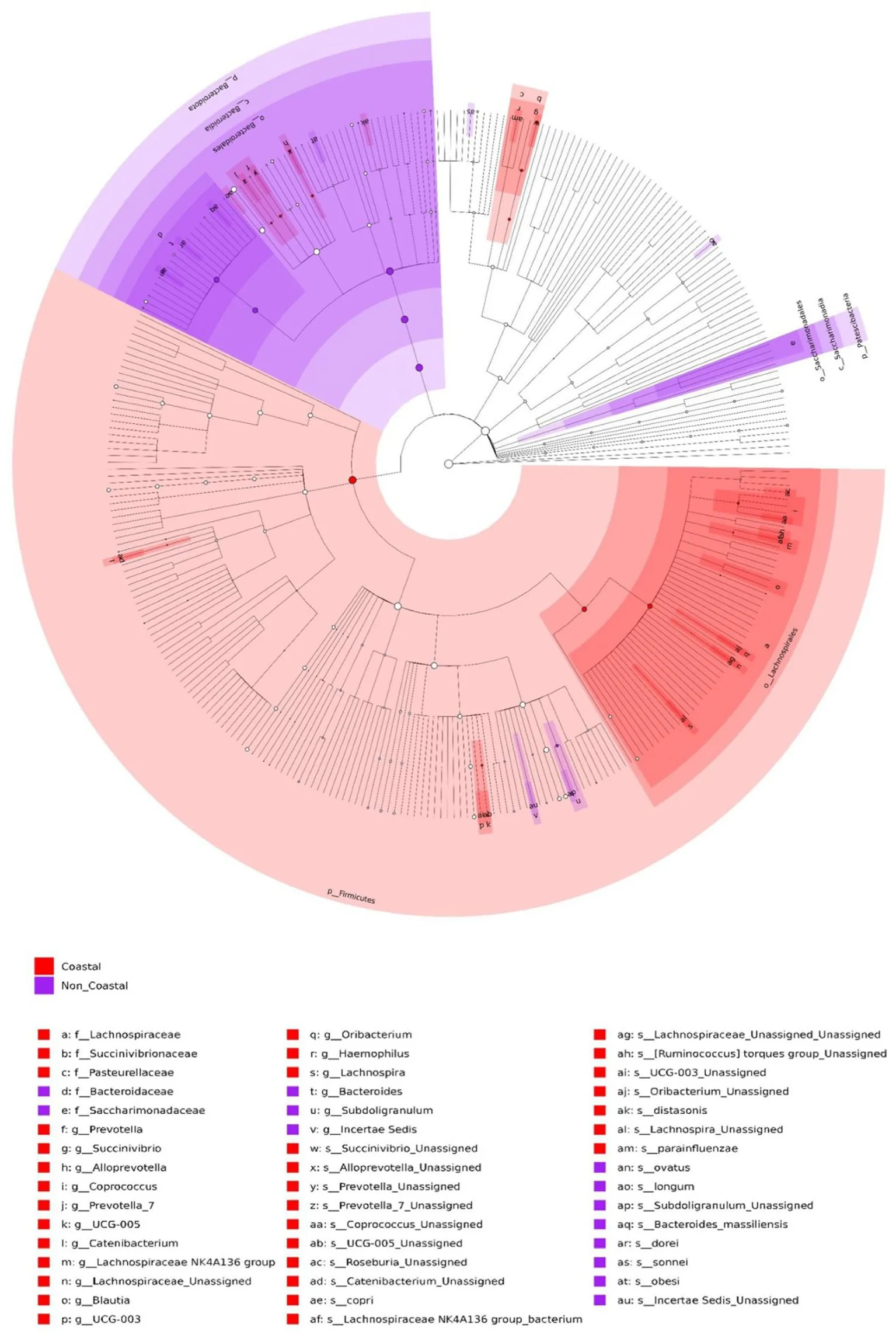
Linear discriminant analysis Effect Size (LEfSe) analysis of the microbial profile of fecal sample between the Coastal and Non-coastal control groups at the phylum-to-order levels (red colour: Coastal group, violet colour: Non-coastal group).

### 3.8 Functional profiling revealed distinct MetaCyc pathways in fecal samples between coastal and non-coastal populations

For each sample, the MetaCyc pathway database by PICRUSt2 was further used to build the functional profiles as shown in Figure 9. The comparison of MetaCyc pathway abundance between coastal and non-coastal samples identified 12 pathways. Based on predicted counts for each pathway, Glycogen Biosynthesis I showed relatively higher abundance in both coastal and non-coastal samples, with comparatively greater representation in coastal samples. Three pathways were predicted to be less expressed, whereas nine pathways were predicted to be enriched based on the log2 fold change. The Super pathway of polyamine biosynthesis II and the superpathway of glycol metabolism and degradation were less expressed, whereas photorespiration was the least expressed. Enriched pathways include the Superpathway of glucose and xylose degradation; pyruvate fermentation to propanoate; pentose phosphate pathway; myo-, chiro-, and scillo-inositol degradation; L-rhamnose degradation I; L-histidine degradation; 4-deoxy-L-threo-hex-4-enopyranuronate degradation; Chondroitin sulfate degradation I; and glycogen biosynthesis I.

**Figure 9:**
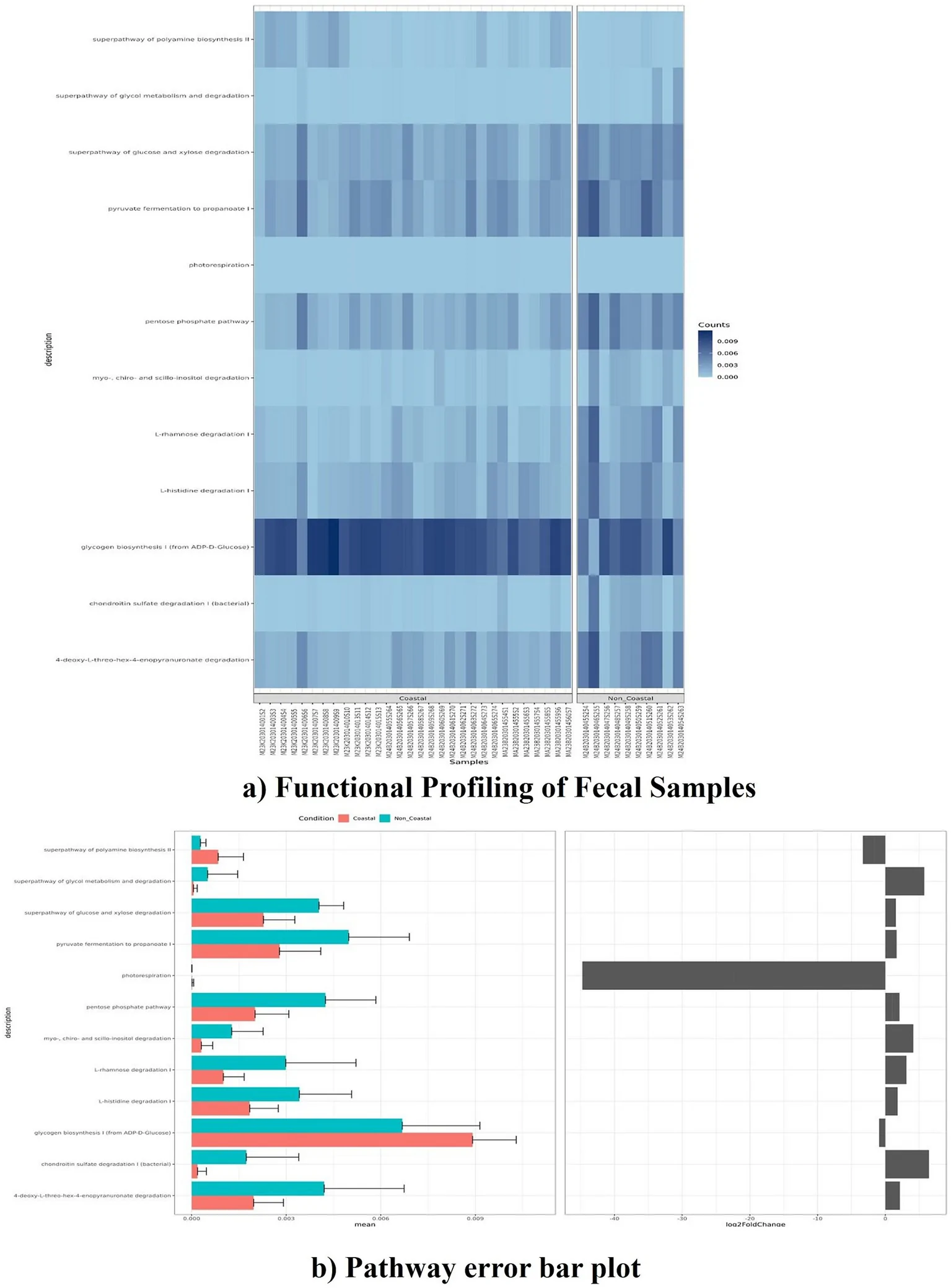
Comparison of the relative abundance of the gut microbiome between coastal and non-coastal groups. **(a)** Functional profiling of fecal samples. **(b)** Pathway error bar plot (red colour: Coastal group, blue colour: Non-coastal group).

## 4 Consequences of gut microbiome dysbiosis in coastal and non-coastal populations

Gut microbiome imbalance has been increasingly associated with the host immune, inflammatory, and metabolic responses (Hrncir, 2022). In the present study, differences in microbial diversity and taxonomic composition were observed between coastal and non-coastal populations. Recent studies suggest that environmental exposure, lifestyle factors, and dietary patterns may influence gut microbial communities (Ahn and Hayes, 2021; Kang et al., 2025). In coastal individuals, the distinct microbial composition and altered relative abundance of beneficial taxa may contribute to alterations in gut microbial homeostasis. Previous studies have suggested that environmental pollutants and heavy metals in certain coastal regions may influence intestinal microbial composition and be associated with alterations in epithelial barrier function and the promotion of chronic inflammation (Harjanti et al., 2024). Recent studies have also indicated that microbiota dysbiosis may be linked to oxidative stress levels (Rezazadegan et al., 2025), modulate cytokine secretion (O’Grady and Grabrucker, 2025), and alter xenobiotic metabolic pathways (Zhu et al., 2024). However, these host physiological and inflammatory parameters were not evaluated in the present study.

The coastal and non-coastal groups exhibited distinct bacterial community profiles, with higher relative abundances of Lachnospiraceae in the coastal group and Bacteroides in the non-coastal group. Previous studies have reported that members of the Lachnospiraceae family are commonly associated with butyrate production, whereas Bacteroides species are associated with propionate production (Kircher et al., 2022). Previous studies have associated these microbial communities with mucosal immune regulation (He and Dong, 2023), improved metabolic stability, and a healthy gut barrier (Nami et al., 2025; Hsu et al., 2024). Moreover, the differences between coastal and non-coastal individuals suggest that coastal individuals may be influenced by environmental factors, such as heavy metal contamination in food and water, which may contribute to increased health risks linked to gut microbial imbalances. Overall, these findings suggest that environmental conditions may help shape gut microbial composition in human populations.

## 5 Discussion

The objective of this pilot study is to examine differences in gut and skin microbiota composition between coastal and non-coastal areas along the East Coast of Tamil Nadu, India. Coastal participants were predominantly involved in fishing-related occupations and reported more frequent seafood consumption compared to non-coastal participants. Lifestyle-related differences, including alcohol consumption and smoking habits, were also observed between the two populations and may contribute to variations in microbiota composition. Findings from the present study revealed that the same dominant microbial phyla identified in previous microbiome studies were also present; however, it compared the gut and skin microbiomes of coastal and non-coastal populations in Tamil Nadu, India. By comparing the coastal and non-coastal groups, we identified the source of variation in microbiome composition and taxonomic distribution between populations exposed to different environmental conditions.

In this study, we analyzed fecal and skin microbiota using 16S rRNA sequencing from participants in non-coastal and coastal groups to explore differences in microbial composition between the two populations. The alpha-diversity analysis of fecal samples using Observed species, Chao1, Shannon, and Simpson indices showed no significant differences between the coastal and non-coastal groups after outlier removal and Benjamini–Hochberg correction. Although some variation in microbial richness and diversity was observed among individual samples, the overall fecal microbial diversity was similar between the two groups. These findings suggest that the differences between coastal and non-coastal populations may be more related to microbial community composition and the relative abundance of specific taxa rather than overall microbial diversity. Although there was partial clustering observed in both PCoA and NMDS beta diversity analyses, overlaps were also observed between non-coastal and coastal participants, highlighting the common core microbiota. PERMANOVA based on Bray–Curtis dissimilarity demonstrated a significant difference in overall microbial community composition between the coastal and non-coastal fecal samples (R^2^ = 5.7%, *p* = 0.001). However, the result suggests that coastal exposure had a limited contribution to the observed variation in microbial composition. Furthermore, the significant multivariate dispersion observed in the fecal samples suggests differences in within-group variability. As PERMANOVA is sensitive to differences in dispersion, these findings should be interpreted with caution, as the observed significance may reflect a combination of differences in community composition and within-group variability. Therefore, the observed alterations should be interpreted as compositional shifts in the microbiota rather than definitive evidence of dysbiosis. Moreover, some comparisons of alpha diversity were not statistically significant after outlier removal in the fecal samples, indicating differences within the groups and underscoring the preliminary nature of the results obtained in the pilot study, whereas the skin swab samples showed statistical significance between the groups after outlier removal. Previous studies have highlighted that Firmicutes and Bacteroidetes are the predominant gut bacterial phyla, and variations in the Firmicutes-to-Bacteroidetes ratio have been associated with alterations in gut microbial composition (Tsai et al., 2024; Mariat et al., 2009). Similarly, a higher relative abundance of Firmicutes was observed in the coastal participants’ fecal samples, whereas Bacteroidota predominated in the non-coastal group. More specifically, coastal samples showed a higher abundance of microbial taxa such as Bacteroides, Lachnospirales, Oscillospirales, and Clostridia UCG-014. These microbial taxa had been previously associated with complex carbohydrate metabolism and short-chain fatty acids. Environmental factors and dietary patterns in coastal regions can influence these compositional differences (Hudson and Egan, 2022). Such compositional shifts may indicate microbial adaptation to environmental stressors and may influence host metabolic and immune functions. Previous studies have reported that a reduced GI microbial community or an imbalance of beneficial microorganisms has been associated with impaired epithelial barrier function, oxidative stress, and inflammatory responses (Harjanti et al., 2024; Rezazadegan et al., 2025; O’Grady and Grabrucker, 2025). Previous studies have suggested that microbiota dysbiosis may influence xenobiotic metabolism or immunoreactive signaling pathways (Zhu et al., 2024), potentially contributing to metabolic or inflammatory diseases. Although such mechanisms have been described in the literature, our findings primarily highlight the differences in microbial composition and taxonomic distribution between coastal and non-coastal populations. These findings provide preliminary insights into microbial variations between coastal and non-coastal populations exposed to different environmental conditions.

In contrast to the fecal microbiome, the skin microbiome showed significant differences in alpha diversity between the coastal and non-coastal groups. After removing the identified outliers and applying the Benjamini–Hochberg (BH) correction, significant differences were observed in Observed OTUs, Chao1, Shannon, and Simpson indices. Since skin conditions such as eczema, tinea, and dry skin were present only in the coastal group, the initial differences may have been influenced by these conditions. Therefore, alpha diversity was also analyzed using only participants from both groups with clinically normal skin. Significant differences remained between the healthy coastal and healthy non-coastal groups after outlier removal and BH correction. This suggests that the observed differences in skin microbial diversity were not explained only by the presence of skin conditions. The significant differences observed among healthy participants suggest that coastal environmental exposure may affect the skin microbiota. Therefore, further studies with balanced samples of healthy and diseased participants are needed to better understand the effects of coastal environmental exposure. Furthermore, beta diversity analysis demonstrated a significant difference in microbial community composition between coastal and non-coastal skin swab samples based on Bray-Curtis PERMANOVA (R^2^ = 3.5%, *p* = 0.041). These findings support differences in skin microbial community composition between the two populations. Although the Firmicutes were dominant in all samples and in both groups, the Bacilli and Clostridia classes were more prevalent in the coastal group. These differences may be associated with environmental exposure, lifestyle factors, and microbial adaptation to coastal conditions. The skin microbiota in PCoA and NMDS analyses was demonstrated through the distribution and overlap between coastal and non-coastal groups. The presence of Firmicutes in skin could be linked to environmental factors and lifestyle variations. Although Firmicutes have been identified as normally found in the skin and gastrointestinal tract, changes in microbiota could be responsible for the emergence of various skin disorders like atopic dermatitis (Hrestak et al., 2022; Totte et al., 2016), acne (Byrd et al., 2018), and psoriasis (Tett et al., 2017). Proteobacteria dominated skin samples due to environmental and geographical factors. A recent study by Potbhare et al. (2022) observed that Firmicutes, Proteobacteria, and Actinobacteria were the prevalent phyla in the skin of genetically unrelated individuals of Indian origin. They also concluded that the geographic location had a major effect on microbial community composition (Potbhare et al., 2022). Additionally, Potbhare et al. (2025) reported that environmental factors (such as geographic location) affect skin microbiota among Indian families, with major variations in bacterial abundance by location (Potbhare et al., 2025). Overall, the gut and skin microbiomes of coastal and non-coastal groups have generally been found to differ in composition, implying that environmental exposure may affect microbial communities across multiple body regions. Although alterations were observed in both the gut and skin microbiota of coastal individuals, the gut and skin microbial communities were analyzed independently. Consequently, it remains unclear whether these changes reflect coordinated host responses across body sites or independent niche-specific adaptations to coastal environmental exposure.

PICRUSt2-based inferences in this study are predictive in nature and require further validation through functional, metabolomic, and biochemical quantifications for establishment of the functional relevance with environmental exposure and host physiological responses. The observed alterations in gut and skin microbiota between coastal and non-coastal populations may be associated with environmental exposures, including pollutants and heavy metals commonly present in coastal regions, which may influence microbial community composition and contribute to shifts in microbial homeostasis, such as increased abundance of opportunistic taxa such as Proteobacteria. Although previous studies have associated such microbial alterations with inflammatory and metabolic disturbances, host physiological and immunological parameters were not evaluated in the present study; therefore, the functional significance of the observed microbial changes remains to be established.

## 6 Limitations and future perspectives

The relatively small sample size and cross-sectional design limit the statistical strength of the findings and restrict the ability to establish relationships between environmental factors and microbiome alterations. As the present study was designed as a pilot exploratory investigation, formal prior sample size or statistical power calculation was not performed before participant recruitment. In addition, unequal sample sizes between the coastal and non-coastal groups may have limited the statistical power and generalizability of the findings. Demographic characteristics, including sex, age, and alcohol consumption, also differed between coastal and non-coastal groups. Since this is a pilot study with a small sample size and unequal distributions of these variables, statistical analyses that adjusted for these factors were not performed. Therefore, these factors may have influenced the observed differences in microbial composition and should be considered when interpreting the findings. Also, the variation in skin conditions between coastal and non-coastal participants (none of the non-coastal participants exhibited skin disease, whereas the coastal group included both normal and diseased skin conditions) may have contributed to the high variability in taxa among the study groups. Therefore, the observed differences in taxa between the groups should not be solely attributed to coastal exposure. Accordingly, the findings should be validated in prospective studies with larger sample sizes. Although fecal microbiota may not accurately represent the mucosal-associated and small-intestinal microbial populations, the fecal samples are widely used because the intestine is the site of greatest colonization and harbors many of the most extensively characterized microorganisms. Additionally, we hypothesized that the relative abundance of bacterial taxa in fecal samples would be used to characterize the gut microbiota.

Future studies that examine the host environment-microbiome interaction at different body sites will assist in enhancing our understanding of the microbial relationship between the gut and skin. Future studies should focus on large populations and functional analyses of the microbiome to understand the complex host-environment-microbiome interaction and its probable role in modulating human health. Moreover, intervention studies aiming to modulate the microbiota with dietary or probiotic therapy, as well as host environmental modifications, may help explore innovative approaches to microbiota modulation for human health tailored to coastal populations.

## 7 Conclusion

This study demonstrates distinct differences in gut and skin microbiota between coastal and non-coastal populations in Tamil Nadu, highlighting the potential influence of environmental exposures, geographical conditions, and lifestyle factors on microbial composition and diversity. The coastal population exhibited a distinct microbial composition and greater differences in alpha and beta diversity than the non-coastal region. Similarly, skin samples from coastal participants exhibited greater richness and distinct microbial profiles, which may reflect variations in environmental exposures, including marine environments, contaminated water sources, seafood-based dietary habits, and occupational activities. The inclusion of participants with skin conditions such as eczema and tinea in the coastal population may also have contributed to variations in skin microbiota composition. The predominance of certain bacterial phyla, such as Firmicutes and Proteobacteria, suggests that environmental factors may influence microbial community structure and microbial homeostasis. Overall, the findings indicate that coastal and non-coastal populations harbor distinct microbial community structures, underscoring the need for further investigation into host-environment microbiome interactions. However, the findings should be interpreted with caution given the comparatively small sample size and the study’s exploratory nature, and therefore require validation in larger and more diverse populations. Furthermore, large-scale studies that integrate metagenomics, functional analyses, environmental assessments, and molecular studies may help clarify the relationship among environmental exposure, microbiome alterations, and human health.

## Data Availability

The datasets presented in this study can be found in online repostories. The 16S rRNA gene sequencing raw data generated in this study have been deposited in the NCBI Sequence Read Archive (SRA) and are publicly available under SRA accession numbers SRR40291314-SRR40291391. The R scripts used for the statistical analysis have now been deposited on GitHub and are publicly available under the repository link https://github.com/PonnaiyasCAG/Metagenomics-Analysis-Pipeline-using-R-script.
