# Supplementary material for: Comparative analysis of gut and skin microbiota from the coastal and non-coastal populations in Tamil Nadu using 16S rRNA gene sequencing: a pilot study
Source: Front Microbiol. 2026 Oct 26;17:1849751. doi: 10.3389/fmicb.2026.1849751 (PMC13745483; doi:10.3389/fmicb.2026.1849751)
Supplement: Supplementary file 1 [file Supplementary_file_1.DOCX]

**Supplementary Table 1:** Demographic characteristics, lifestyle factors, and dietary patterns of coastal and non-coastal participants included in fecal and skin swab microbiome analysis.

| **Characteristics** | **Fecal samples** | | **Skin swab samples** | |
| --- | --- | --- | --- | --- |
|  | **Non-coastal population** | **Coastal population** | **Non-coastal population** | **Coastal population** |
|  | **n=10 (%)** | **n=30 (%)** | **n=10 (%)** | **n=28 (%)** |
| **Age** | | | | |
| 19-59 years | 10 (100) | 24 (80) | 10 (100) | 20 (71.4) |
| **≥** 60 years | **-** | 6 (20) | - | 8 (28.6) |
| **Gender** | | | | |
| Male | 9(90) | 9(30) | 9 (90) | 16 (57.1) |
| Female | 1(10) | 21(70) | 1 (10) | 12 (42.9) |
| **Occupation** | | | | |
| Housewife | 1 (10) | 17 (56.7) | 1 (10) | 5 (17.9) |
| Fishermen/women | - | 9 (30) | - | 16 (57.1) |
| Industrial/Mining sector | 6 (60) | - | - | - |
| Technical/Administrative | 2 (20) | - | 8 (80) | - |
| Security service | 1 (10) | - | 1 (10) | - |
| Retired | - | 2 (6.7) |  | 1 (3.6) |
| *Others |  | 2 (6.6) | - | 6 (21.4) |
| *Others - Tailor, Teacher, Shopkeeper, Welder, Driver, Housekeeping | | | | |
| **Alcohol consumption** | | | | |
| Yes | 4 (40) | 27 (90) | 4 (40) | 11 (39.3) |
| No | 6 (60) | 3 (10) | 6 (60) | 17 (60.7) |
| **Smoking habit** | | | | |
| Yes | 1 (10) | - | 3 (30) | 1 (3.6) |
| No | 9 (90) | 30 (100) | 7 (70) | 27 (96.4) |
| **Dietary pattern** | | | | |
| Vegetarian | 1 (10) | 3 (10) | 3 (30) | 2 (7.1) |
| Non-vegetarian | 9 (90) | 27 (90) | 7 (70) | 26 (92.9) |
| **Seafood consumption** | | | | |
|  | **n=9 (%)** | **n=27 (%)** | **n=7 (%)** | **n=26 (%)** |
| Regular | 4 (44.4) | 22 (81.5) | 3 (42.9) | 23 (88.5) |
| Occasional | 5 (55.6) | 2 (7.4) | 4 (57.1) | 3 (11.5) |
| No seafood | - | 3 (11.1) | - | - |

**Supplementary Table 2:** Comparison of alpha diversity indices (Observed species, Chao1, Shannon, and Simpson) in fecal samples from coastal and non-coastal groups. Statistical analysis was performed using the Mann-Whitney U test, with p-values adjusted for multiple comparisons using the Benjamini–Hochberg (BH) method. **U-value** - Mann-Whitney U statistic; **q-value** - Benjamini-Hochberg (BH) adjusted p-value.

| **Index** | **Median**  **(Non-coastal)** | **Median**  **(Coastal)** | **U-value** | **p-value** | **q-value** |
| --- | --- | --- | --- | --- | --- |
| **Observed** | 33.00 | 49.00 | 87.5 | 0.1441 | 0.2882 |
| **Chao1** | 32.00 | 49.00 | 87.5 | 0.0648 | 0.2592 |
| **Shannon** | 2.65 | 3.02 | 94.0 | 0.2701 | 0.3601 |
| **Simpson** | 0.89 | 0.92 | 93.0 | 0.3817 | 0.3817 |

**Supplementary Table 3:** Distribution of skin conditions among coastal and non-coastal skin swab participants.

| **Skin conditions** | **Non-coastal population** | **Coastal population** |
| --- | --- | --- |
|  | **n=10 (%)** | **n=28 (%)** |
| Normal skin | 10 (100) | 15 (53.6) |
| Eczema | - | 7 (25) |
| Tinea | - | 5 (17.9) |
| Dry skin | - | 1 (3.5) |

**Supplementary Table 4a:** Comparison of alpha diversity indices (Observed species, Chao1, Shannon, and Simpson) in skin swab samples from coastal and non-coastal groups. Statistical analysis was performed using the Mann-Whitney U test, with p-values adjusted for multiple comparisons using the Benjamini–Hochberg (BH) method. **U-value** - Mann-Whitney U statistic; ***** - p-value ≤ 0.05; ****** - p-value ≤ 0.01; ******* - p-value ≤ 0.001; **q-value** - Benjamini-Hochberg (BH) adjusted p-value.

| **Index** | **Median**  **(Non-coastal)** | **Median**  **(Coastal)** | **U-value** | **p-value** | **q-value** |
| --- | --- | --- | --- | --- | --- |
| **Observed** | 19.50 | 47.50 | 40.5 | 0.0009*** | 0.002 |
| **Chao1** | 21.00 | 47.87 | 41.0 | 0.001** | 0.002 |
| **Shannon** | 2.11 | 2.89 | 64.0 | 0.0276* | 0.028 |
| **Simpson** | 0.80 | 0.91 | 50.0 | 0.0181* | 0.024 |

**Supplementary Table 4b:** Comparison of alpha diversity indices (Observed species, Chao1, Shannon, and Simpson) in skin swab samples from healthy coastal and healthy non-coastal groups. Statistical analysis was performed using the Mann-Whitney U test, with p-values adjusted for multiple comparisons using the Benjamini–Hochberg (BH) method. **U-value** - Mann-Whitney U statistic; ***** - p-value ≤ 0.05; ****** - p-value ≤ 0.01; **q-value** - Benjamini-Hochberg (BH) adjusted p-value.

| **Index** | **Median**  **(Non-coastal)** | **Median**  **(Coastal)** | **U-value** | **p-value** | **q-value** |
| --- | --- | --- | --- | --- | --- |
| **Observed** | 19.50 | 47.00 | 20.50 | 0.0064** | 0.0036 |
| **Chao1** | 21.00 | 47.60 | 21.00 | 0.0036** | 0.0036 |
| **Shannon** | 2.11 | 2.87 | 29.00 | 0.0212* | 0.0212 |
| **Simpson** | 0.80 | 0.91 | 19.00 | 0.0095** | 0.0095 |
